# Sediment sampling with a core sampler equipped with aluminum tubes and an onboard processing protocol to avoid plastic contamination

**DOI:** 10.1016/j.mex.2019.10.027

**Published:** 2019-11-01

**Authors:** Masashi Tsuchiya, Hidetaka Nomaki, Tomo Kitahashi, Ryota Nakajima, Katsunori Fujikura

**Affiliations:** Japan Agency for Marine-Earth Science and Technology (JAMSTEC), 2-15 Natsushima-cho, Yokosuka 237-0061, Japan

**Keywords:** Non-plastic-contaminated sediment core sampler, Microplastics, Aluminum-made core sampler, Environmental monitoring

## Abstract

Microplastics are abundant even on the deep-sea floor far from land and the ocean surface where human activities take place. To obtain samples of microplastics from the deep-sea floor, a research vessel and suitable sampling equipment, such as a multiple corer, a box corer, or a push corer manipulated by a remotely operated (ROV) or human occupied vehicle (HOV) are needed. Most such corers use sampling tubes made of plastic, such as polycarbonate, acrylic, or polyvinyl chloride. These plastic tubes are easily scratched by sediment particles, in particular during collection of coarse sandy sediments, and, consequently, the samples may become contaminated with plastic from the tube. Here, we report on the use of aluminum tubes with both a multiple corer and a push corer to prevent such plastic contamination. When compared with plastic tubes, aluminum tubes have the disadvantages of heavier weight and non-transparency. We suggest ways to overcome these problems, and we also present an onboard processing protocol to prevent plastic contamination during sediment core sampling when plastic tubes are used.

• Use of a sediment corer with aluminum tubes reduces the risk of plastic contamination in the sediment samples
• The proposed method allows undisturbed sediment cores to be retrieved with comparable efficiency to conventional transparent core tubes

## Graphical abstract

**Figure:**
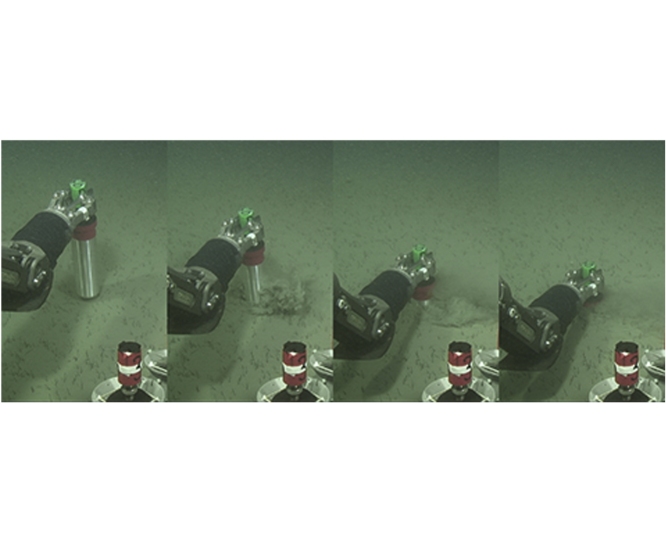

**Specification Table**

**Table:** 

Subject Area:	Environmental Science
More specific subject area:	Microplastic monitoring
Method name:	non-plastic-contaminated sediment core sampler
Name and reference of original method:	Sediment core sampler
Resource availability:	Not available

## Method details

**Fig. 1:**
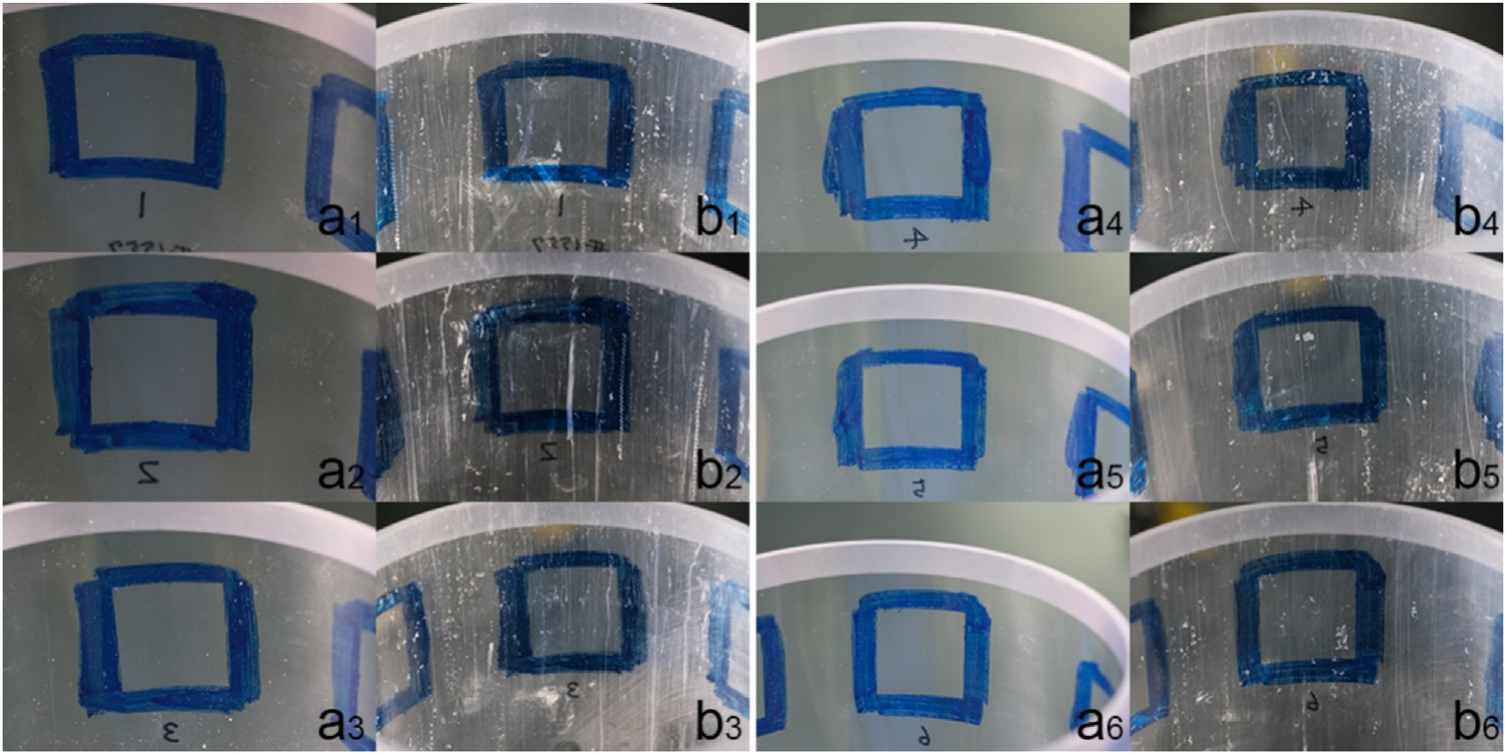
**A polycarbonate pipe for a push corer exhibiting abundant scratches after sediment samplings.** Six areas were selected from a polycarbonate tube. Square indicates 1 × 1 cm area. a: inner wall of a new polycarbonate core tube; b. after retrieving a core (HOV *Shinkai 6500*, Dive #1557, YK19-11, at a depth of 855 m, 35˚0.9540′N,139˚13.3250′E).

We describe methods for collecting sediments with a push corer and a multiple corer equipped with aluminum sampling tubes to minimize the possibility of plastic contamination. Typically, these coring devices are equipped with sampling tubes made of plastic such as polycarbonate, acrylic, or polyvinyl chloride. However, these plastic tubes, which can be easily scratched by sediment particles and melted at the hydrothermal vent fields, in particular during collection of sandy sediments, are a potential source of plastic contamination of sediment samples (Fig. 1). The use of an aluminum tube can reduce these potential contamination risks. The advantages of aluminum over other metals is that it is easy to shape, light, relatively robust, and inexpensive. All these tubes were designed to the same dimensions as conventional polycarbonate or acrylic tubes for a multiple corer or a push corer, except for a small modification to the inner diameter of the push core. We introduce some examples of sediment sample collections using these sampling gears on the deep-sea floor and discuss some advantages and disadvantages to the method. To clarify the spatio-temporal distribution of microplastics, it is necessary to accurately analyze their vertical distribution in sediments, and this requires the collection of undisturbed sediment samples.

**Fig. 2:**
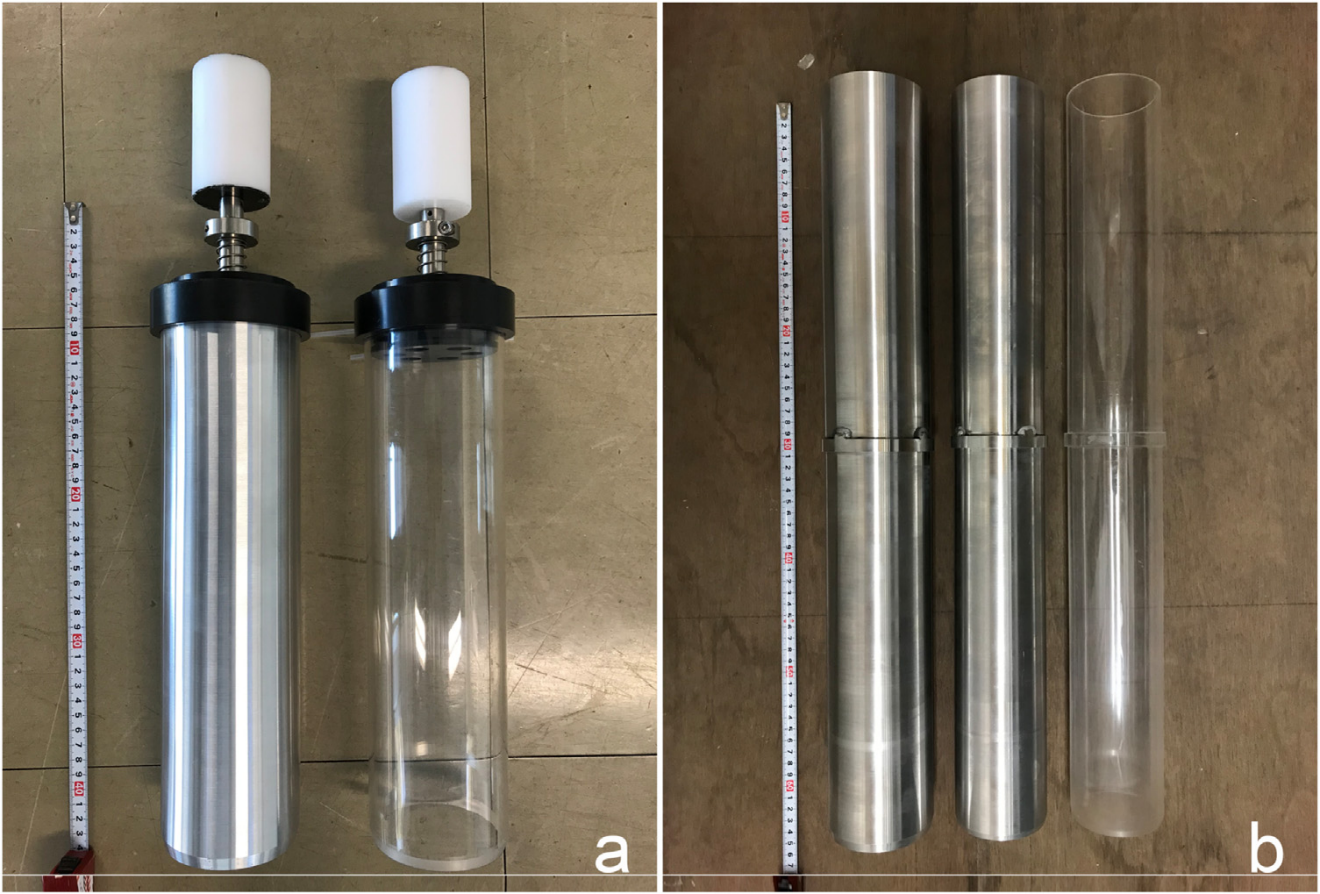
**Aluminum pipes for a) a push corer and b) a multiple corer.** Both tubes have an inner diameter of 82 mm and an outer diameter of 89 mm, which is the same size as the conventional push corers used at JAMSTEC and the multiple corer belonging to the Atmosphere and Ocean Research Institute, University of Tokyo.

A major problem arise during sediment collection and processing: contamination from the surrounding environment [1], particulary from the inner wall of plastic core tube. We therefore designed and tested methods for addressing these problems (Fig. 2).

We designed and tested core tubes made of aluminum for both a multiple corer (74 mm or 82 mm inner diameter and 600 mm in length) and a push core (82 mm inner diameter and 320 mm in length) with the aim to avoid contamination by microplastics during deep-sea sediment sampling, particularly in areas with coarse sediments (Fig. 2). Sampling tubes made of polycarbonate or acrylic have the advantage of being transparent, allowing the sampled sediments to be observed from the outside.

Contamination can also occur when sediment samples are processed. For example, a core extruder is typically used when a sediment core needs to be sliced at certain intervals without disturbing the sediment layers [2]. If the core extruder is made of PVC, then the inner wall of the sampling tube can be damaged by sand grains, just as it can be when the sediment core is collected, and microplastic contamination may occur. To reduce the possibility of plastic contamination, we used a core extruder with an aluminum head (the part that comes into contact with the sampling tube) (Fig. S1). Similarly, a metal cutting plate can be used to slice the core sediments into layers.

## Tests at the deep-sea floor

Sediment samplings using the aluminum-made tubes were performed during the KS18-J02 cruise in March 2018, the YK19-11 cruise in September 2019, and the KM19-07 cruise in September 2019 in Japanese waters. The water depth ranged from 855 to 9232 m and retrieved cores from fine sand to silt sediments.

During the KS18-J02 cruise, the push coring using aluminum tubes was carried out with the ROV *Hyper-Dolphin*. During the YK19-11 cruise, the push coring was carried out by the HOV *Shinkai 6500*. Push cores were installed in a push core holder made with PVC, but the bottom of the holder was covered by a 5 mm-thick aluminum plate, preventing the bottom of the sediments coming into contact with plastic materials.

**Fig. 3:**
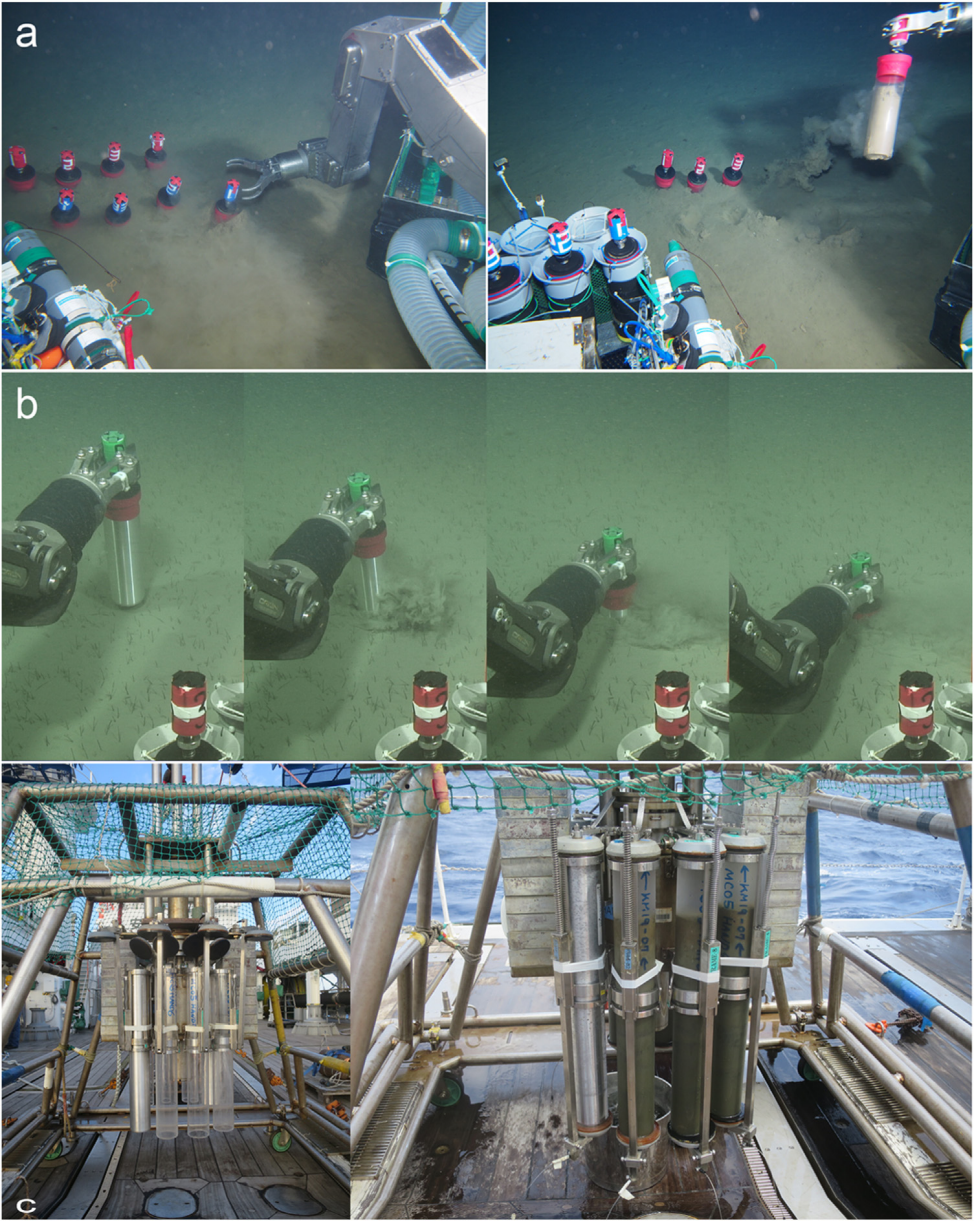
**Push core sampling at the deep-sea floor.** a: Aluminum tube push core (front-row cores) used together with the conventional polycarbonate tube push corer (back-row cores) at the deep-sea floor (HOV *Shinkai 6500*, Dive #1553, at a depth of 5719 m, 33˚0.2359′N,145˚0.7300′E); b: Aluminum tube push core sampling at the deep-sea floor (ROV *Hyper-Dolphin*, Dive #2041, at a depth of 1548 m, 34˚54.8968′N 138˚39.0413′E). c: A multiple corer equipped with both aluminum tubes and conventional polycarbonate tubes on board.

Push core samplings were conducted using a corer with an aluminum sampling tube and found that the major problem was the non-transparency of the tube, which made it difficult to judge how deep the corer had penetrated and how much sediment was retained within the coring tube during its retrieval. Therefore, we used a push corer with a conventional polycarbonate tube for the first trial (Fig. 3). Thus, we were able to determine from the polycarbonate core sampling results the hardness of the sediment, how deep we needed to insert the aluminum sampling tube, and the necessary recovery manipulation. We were able to refer to the results of the polycarbonate core samplings with respect to how deep the corer needed to be inserted and how soft or hard the sediment was, assuming that friction between the sediments and aluminum or polycarbonate are similar.

Another problem is sediment disturbance. As described above, it is important not only to collect microplastics but also to analyze their vertical distribution within the sediment layers collected, without disturbing the sediment structure. When samples are collected with push corers or multiple corers with aluminum tubes, the collected sediment cannot be seen from the outside. In particular, when a submersible is used for core collection, the operation must be conducted with a manipulator and the depth of insertion into the sediment depends on sediment hardness and particle size. Thus, it is desirable to be able to see the inside of the tube in order to gauge the insertion depth (Fig. 3). If push coring is being conducted by a submersible, a corer with a polycarbonate sampling tube can be inserted into the sediment at the same time as one with an aluminum tube and the insertion depth of the latter can be adjusted by comparison with that of the former. Similarly, when a multiple corer is used, it is possible to estimate the approximate insertion depth by installing tubes made of polycarbonate as well as ones made of aluminum.

We next conducted tests with a multiple sampler equipped with both aluminum and polycarbonate sampling tubes. During the KM19-07 cruise, two out of eight tubes were replaced with aluminum-made tubes. When we deployed the aluminum cores on multiple corer, the major problem was an imbalance between the heavier aluminum tubes and the lighter polycarbonate tubes. This imbalance could cause the multiple corer to tilt and lead to uneven penetration of the sediment by the tubes. Another problem encountered was premature triggering of the device before recovery due to the load on the central axis of the multiple corer. We thus placed the two aluminum tubes to be diagonally opposite each other.

After retrieving the cores onboard, they were placed on the core extruder [2] with the aluminum-plate on the bottom. Sediment samples were sliced horizontally into desired vertical depths (some mm to cm). Overlying water was removed with a siphon tube. However, again, due to the non-transparency of the core, it was difficult to confirm the position of the sediment-water interface through the tube. We therefore needed to illuminate the top of the core with a flashlight to confirm the position of the sediment-water interface so as not to suck off the fluffy surface sediments (Fig. S2).

Previous studies using plastic tubes for microplastic collections in the sediments trimmed the periphery of the sediment core attached to the tube (e.g. 1 cm in width) as quantitatively as possible to avoid contamination [3] (Fig. S3). However the trimming of the peripheral part of the sliced sediments (1 cm in width) of widely-used cores (e.g. inner diameter of 82 mm) can result in 43 % loss of the sediments. For deep-sea sediment where there is a limited chance for sampling and a low number of retrieved cores during scientific cruises and /or submersible dives, we recommend the use of a corer with an aluminum tube for sampling of microplastics.

The sliced sediment samples were put into glass bottles, which were pre-combusted at 450 °C for 3 h, and then sealed with aluminum foil on top. All these samplings were carried out with blank bottles that were placed in the sampling area. To avoid microplastic contamination from the ambient laboratory environment, we used a clean bench [1] and wore lab coats made of cotton with a static protection cover during experiments on the ship. If a clean bench is not available onboard it is necessary to be extremely careful of contamination and do several blank runs as appropriate.

## Conclusions

To avoid plastic contamination from the core, we designed core samplers with aluminum sampling tubes and tested them to collect undisturbed sediment samples from the deep-sea floor. In addition, by attaching an aluminum head to the core extruder, we reduced the risk of microplastic contamination of the sediment samples. Although aluminum tubes have some disadvantages, such as heavier weight and non-transparency, this reduces contamination risks from plastics during sample collection and processing, and has advantages over trimming the core periphery after retrieving the sediment samples when using a plastic tube.

## Additional information

Plastic products that end up in the environment eventually fragment into microplastics, particles 5 mm or less in size, by photo- and thermal degradation, as well as through physical abrasion due to wave action, collisions with sand particles, etc. [4]. Microplastics are ubiquitous across marine and freshwater environments in the surface water, water column and the sediments, with sediments being thought to possibly be the biggest sink of plastics. Of the plastic that enters the ocean, some of the missing plastic [5] may be floating on the sea surface [6], or in the ocean [7] but where most of the plastic has gone is still unknown. One possible destination of marine microplastics is bottom sediments.

Seafloor sediments are sampled by inserting sampling tubes directly into the sediments. This is usually done by a scuba diver in shallow waters and, for the deep-sea floor, various types of sediment samplers can also be deployed from research vessels, including multiple corers, box corers, piston corers, gravity corers, Phleger corers, Ekman-Birge sediment samplers, and Smith-Mcintyre bottom samplers. A multiple corer, or a push corer inserted by the manipulator arm of a remotely operated vehicle (ROV) or a human occupied vehicle (HOV), can retrieve undisturbed sediment samples.

Microplastic pollution has been reported from almost all marine environments [8]. Even in pelagic waters, considerable amounts of microplastics have been detected, particularly in the subtropical gyres [9]. There is less information on the distribution of microplastics in sediments, particularly in deep-sea sediments [reviewed in 10]. Although the deep-sea floor is remote from human activities and had been thought to be less contaminated by microplastics, some studies have reported considerable amounts of microplastics in deep-sea sediments [10]. Furthermore, deep-sea sediments are less disturbed than shallow waters by dredging, storm water discharge, landslides, and so on, and are thus expected to have the potential to be used for reconstructing the deposition histories of microplastics through time, based on precise age reconstructions using radionuclides [11].

Sampling of deep-sea sediments requires some sampling gears that are specifically designed for deep-sea areas, such as a multiple corer, a box corer, a grab corer, and/or a push corer operated by a HOV or a ROV. Among those sampling gears, both the multiple corer and the push corer are known to minimize the disturbance of surface sediments during sediment sampling. Therefore these two sampling methods are useful for studies on microplastic distribution in sediments.

The above sampling gears use tubes made of plastic to collect sediments – typically either acryl or polycarbonate. During both sampling at the deep-sea floor (i.e. insertion of plastic tubes into sediments) and sediment extrusion from the tube on board [see 6], these plastic tubes are scratched by hard sediment particles, particularly at sandy sediment sites (Fig. 1). The scratching produces small plastic particles originating from the sampling tubes, leading to contamination of sediment samples.

The plastic corers can still used for other sampling. To collect undisturbed sediment core, the use of transparent plastic tube is advantageous. Undisturbed samples can also be used to clarify material cycling in the sediment surface and near-bottom layer. So that past and present environments recorded in the sediments can be inferred and sediment age can be correctly estimated, sediment samples need to be collected without disturbing the sedimentary structure. For example, analyses of the meiobenthos (foraminifera, nematodes, copepods, etc.) and microbes (bacteria and virus), and their vertical distribution in seafloor sediments can reveal seasonal fluctuations in the benthic environment. Furthermore, analyses of sediment physical properties (e.g., sediment structures, dissolved oxygen content, *p*H, nutrient contents, and ^210^Pb and ^14^C contents) allow estimation of the environment at the time of deposition and the sedimentation age.

Although we know the material the plastic sampling tube is made of and can thus speculate that particular plastic materials in sediment samples may originate from the core tube, it is better to use non-plastic sampling gear.
