# Supplementary material for: Sediment sampling with a core sampler equipped with aluminum tubes and an onboard processing protocol to avoid plastic contamination
Source: MethodsX. 2019 Nov 1;6:2662–8. doi: 10.1016/j.mex.2019.10.027 (PMC6883351; doi:10.1016/j.mex.2019.10.027)
Supplement: Supplementary file 1 [file mmc1.pdf]

Sediment sampling with a core sampler equipped with aluminum tubes and an onboard processing protocol to avoid plastic contamination

Masashi Tsuchiya*, Hidetaka Nomaki, Tomo Kitahashi, Ryota Nakajima, Katsunori Fujikura

Japan Agency for Marine-Earth Science and Technology (JAMSTEC), 2-15 Natsushima-cho, Yokosuka 237-0061, Japan

*Corresponding author
Email address: tsuchiyam@jamstec.go.jp (Masashi Tsuchiya)

Supplemental figures

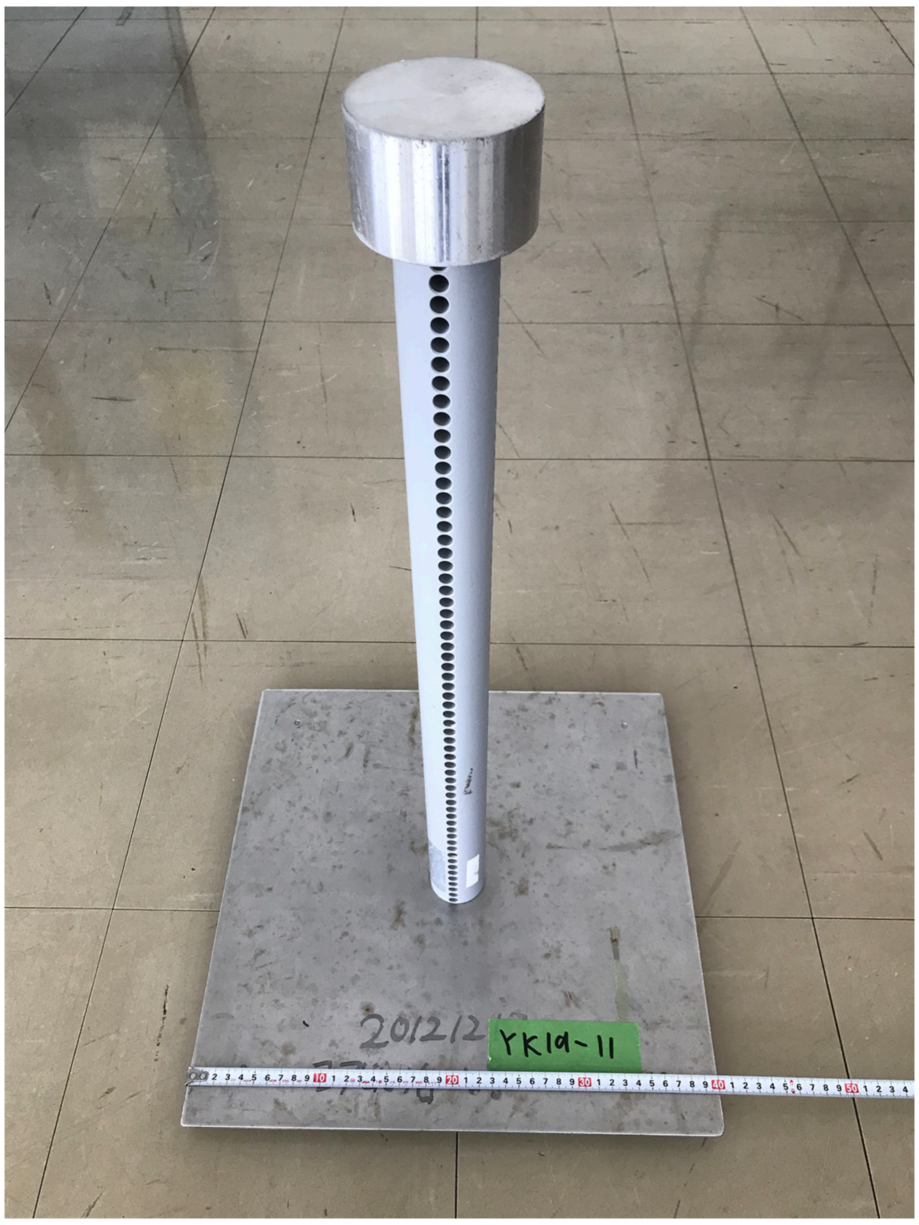

Fig. S1. A core extruder with an aluminum head for slicing the sediment.

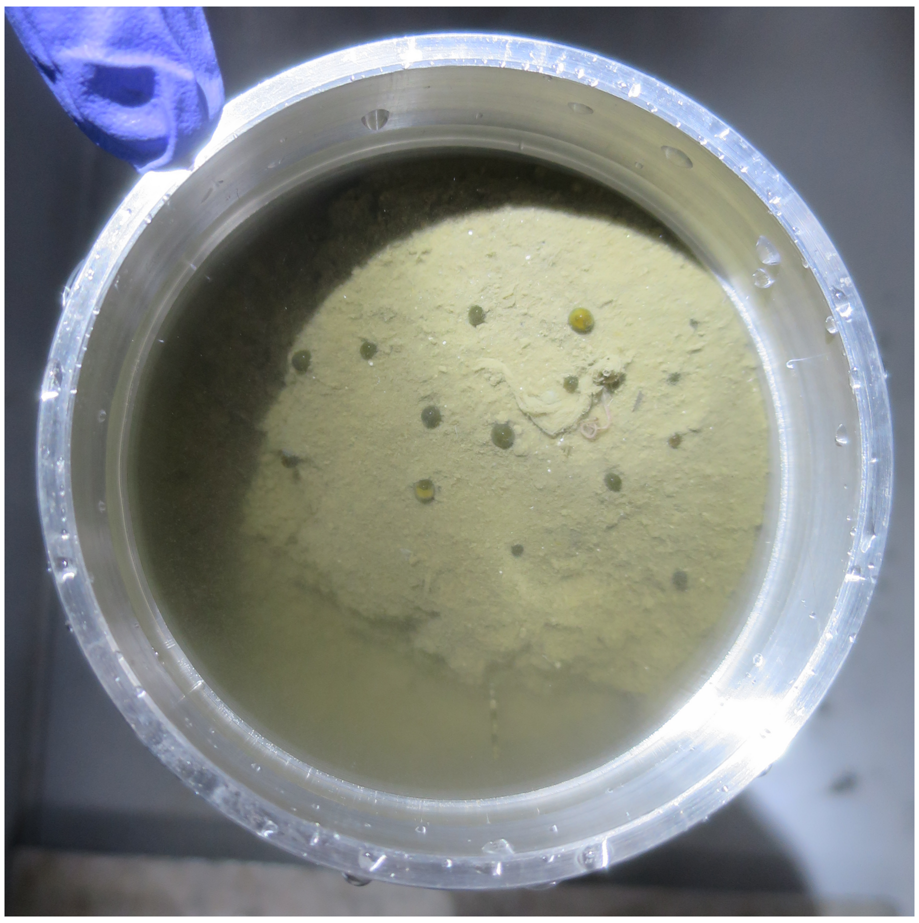

Fig. S2. Collected sediment without disturbing surface. Black dots are unicellular microbial eukaryotes (*Gromia* sp.) that live on the surface of sediment (ROV *Hyper-Dolphin* Dive #2044, KS18-J02, at a depth of 3595 m, 34°11.2328'N 138°25.3218'E).

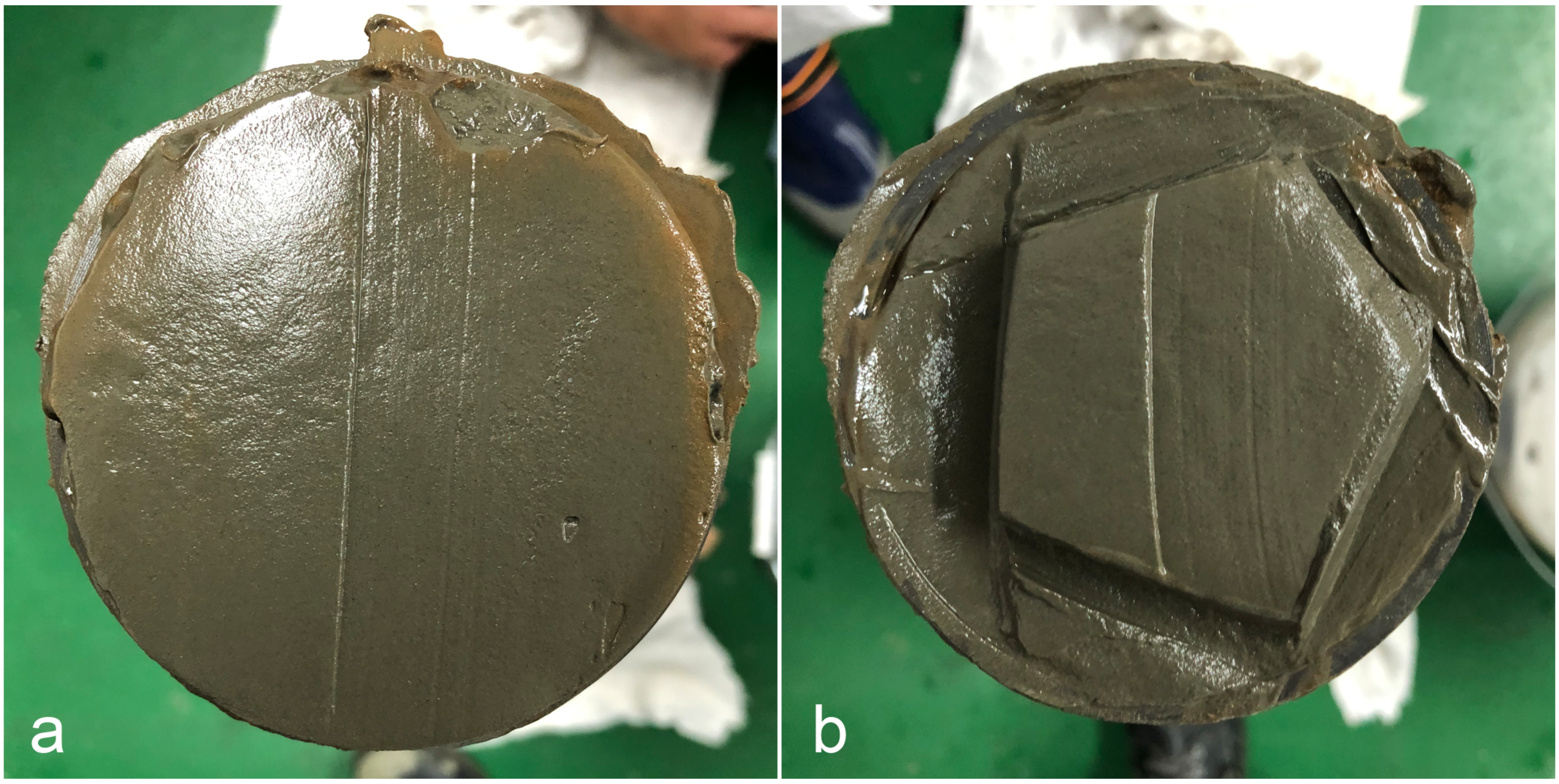

Fig. S3. Trimming of the periphery of sediment core. a: extruded sediment core from core tube; b: trimmed the periphery of the sediment core.
